# PD36 - Evaluation of intracellular cytokines IL-2, IFNγ, IL-4 and IL-5 in children with atopic dermatitis and correlations with other immunological and epidemiological parameters

**DOI:** 10.1186/2045-7022-4-S1-P36

**Published:** 2014-02-28

**Authors:** Maria Koulouri, Efie Vrachnou, Emmanouil Liatsis, Andreas Konstantopoulos, Maria Kanariou

**Affiliations:** 1 Department of Immunology and Histocompatibility, “Aghia Sophia” Children’s Hospital, Athens, Greece; 2 1st Pediatric Clinic, University of Athens, Athens, Greece

## Introduction

Atopic dermatitis is a chronic inflammatory disorder of the skin, charachterised by a TH2 skewed cytokine profile, usually elevated total serum IgE and can be co coexpressed with other atopic clinical manifestations. The atopic skin is prone to bacterial colonization and that has an effect on immunological parameters. Atopic Dermatitis has been correlated with IgG hypoglobulinaemia, low serum IgA and downregulation of the CD8 lymphocytes in peripheral blood.

## Scope

The aim of the study was to evaluate intracellular Th1 and Th2 cytokines secreted by CD3 T cells in children with Atopic Dermatitis, irrespectively of the coexpression of other atopic manifestations and their correlation with other immunological parameters regarding innate and adaptive immunity. Secondly, we aimed to consider the impact of epidemiological characteristics of the patients to the above mentioned immunological values.

## Methods

Peripheral blood samples were collected from 37 children from 3 months to 16 years of age (mean age = 18 months), suffering from Atopic Dermatitis. The percentage of CD3 T cells expressing IL-2, IFNγ, IL-4 and IL-5 was assessed upon phorbol 12-myrisate/ ionomycin stimulation, in the presence of monensin, by flow cytometry. We also measured the concentration of serum Immunoglobulins, total IgE, RASTs and serum concentrations of the above mentioned cytokines.

## Results

We noticed that the percentage of intracellular IL-5 expression was analogous to the atopic bargain of each patient but also to the percentage of CD8 cytotoxic cells in peripheral blood.The severity of Atopic Dermatitis seemed to be inversely correlated to the small age, the male gender and the bacterial skin infection. The total IgE was elevated in most of the patients and had no relation with disease severity. There was a significant correlation between intracellular IFNγ and the female gender. The serum cytokines didn't seem to have similar correlations with their intracellular counterparts.

## Synopsis

The TH2 cell-mediated immune response seems to be pivotal in Atopic Dermatitis pathogenesis. Many other factors affect the immune response, like the gender, the age, the concommitant expression of another atopic phenotype or the bacterial skin infection. The TH1/TH2 concept seems to be intensely affected by the role of TH1 cytokine IFNγ and the TH2 cytokine IL-5, which play an important role to disease dysregulation and the propensity to skin infections.

